# Temporary Mechanical Support in Cardiogenic Shock Secondary to Heart Failure: An Evolving Paradigm

**DOI:** 10.3390/jpm15050184

**Published:** 2025-05-02

**Authors:** Nandini Nair, Dongping Du, Balakrishnan Mahesh

**Affiliations:** 1 Division of Cardiology, Department of Medicine, PSU College of Medicine, Hershey, PA 17033, USA; 2 Industrial, Manufacturing, Systems Engineering, Texas Tech University, Lubbock, TX 79409, USA; dongping.du@ttu.edu; 3 Division of Cardiothoracic Surgery, Department of Surgery, PSU College of Medicine, Hershey, PA 17033, USA; bmahesh@pennstatehealth.psu.edu

**Keywords:** cardiogenic shock, cardiogenic shock outcomes in women, etiology of cardiogenic shock

## Abstract

Cardiogenic shock can be defined as a state of circulatory collapse resulting in hypoperfusion and end-organ dysfunction. It carries a large burden of mortality, but management strategies are driven by expert consensus rather than adequately powered randomized clinical trials. The goal of this review is to highlight the differences in presentation and outcomes in cardiogenic shock depending on the etiology, such as acute myocardial infarction (AMI) versus acute-on-chronic heart failure (HF), gender-based differences in treatment strategies and outcomes and the need for more precise risk stratification and modeling to improve the efficiency of treatment delivery in a personalized fashion. PubMed and Google Scholar were used to search the literature for this qualitative review. The differences in gender and etiology of cardiogenic shock are not consistent in all studies in the exiting literature. There is a need for identification of novel risk factors that define the different phenotypes that present with similar hemodynamic and biomarker profiles. There is an urgent need to devise a methodology to understand and differentiate the different cardiogenic shock phenotypes and their trajectories. Better risk prediction models should be generated to help deliver well-tailored treatment, paving the way to the efficient delivery of personalized medicine.

## 1. Introduction

Cardiogenic shock (CS) can be defined as a state of circulatory collapse resulting in hypoperfusion and end-organ dysfunction. CS carries a large burden of mortality but management strategies are driven by expert consensus rather than adequately powered randomized clinical trials. CS can be multifactorial, with short-term mortality estimated as approaching approximately 50%. The use of temporary mechanical circulatory support (tMCS) in CS has increased dramatically despite a lack of robust randomized controlled trials or evidence suggesting any significant improvement in mortality.

Many factors play into the success of therapeutic strategies, such as timing, choice of therapy based on etiology of CS, appropriate escalation and de-escalation of therapies, transitioning from one device to another at the right time and for the right patient and the use of de-escalation strategies as a bridge to recovery or other durable long-term therapies, such as durable left ventricular assist device (LVAD) implantation or cardiac transplantation. In the event of an unsuccessful attempt to bridge to other therapies, there needs to be an exit strategy including referral to palliative and/or hospice care. Therefore, efficient risk stratification and early interventions should drive management to improve outcomes [1,2].

The goal of this review is to highlight the differences in presentation and outcomes in CS depending on the etiology, such as acute myocardial infarction (AMI) versus acute-on-chronic heart failure (HF), gender-based differences in treatment strategies and outcomes and the need for more precise risk stratification and modeling to improve the efficiency of treatment delivery in a personalized fashion. The use of artificial intelligence (AI)-based technologies to identify risk factors and develop risk prediction models with robust discriminatory power will possibly help improve outcomes and tailor therapy on an individualized basis.

## 2. Materials and Methods

PubMed and Google Scholar were used to search the literature for this qualitative review from 1995 to 2024. Papers published in peer-reviewed journals identified using appropriate key words were analyzed qualitatively, and information was synthesized to arrive at the conclusions presented in this review. The keywords used were cardiogenic shock, etiologies for cardiogenic shock, gender-based differences and cardiogenic shock outcomes.

## 3. Results

### 3.1. MCS Use in Different Stages of Cardiogenic Shock

**Figure 1:**
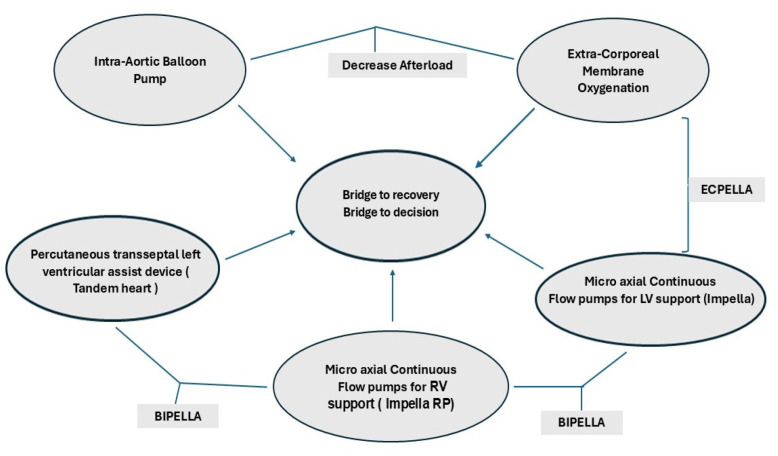
TMCS and different configurations for biventricular support.

Temporary mechanical circulatory support (tMCS) use in cardiogenic shock has increased tremendously despite a lack of evidence-based clinical decision-making with very limited guidelines on tMCS utilization, escalation and de-escalation. Current recommendations are therefore based on expert consensus with respect to treatment strategies and not based on randomized clinical trials [3]. The tMCSs currently available include a whole band of devices starting with the intra-aortic balloon pump (IABP), micro-axial continuous flow pumps for support of the left and right ventricle (Impella), percutaneous trans-septal left ventricular assist device (Tandem heart) and extracorporeal membrane oxygenation (ECMO) as a bridge to recovery or bridge to decision, as shown in Figure 1.

tMCS can be used to increase end-organ perfusion in patients with de novo or refractory CS. It can provide short-term hemodynamic support up to a few weeks and may be used as a bridge to a long-term alternative, such as LVAD or cardiac transplantation. tMCS can be percutaneous or surgical with centrally placed connections to extracorporeal devices or hybrid devices that provide partial or full support to accomplish differential requirements for myocardial oxygen consumption and unloading of the left ventricle, such as the “ECPELLA”, where an ECMO circuit is placed along with a micro-axial pump (Impella) for decompression of the LV. A VA-ECMO can be used also with an intra-aortic balloon pump (IABP). However IABP only reduces the afterload, and the unloading of the left ventricle may not be as effective as using a micro-axial pump. Other variations, such as a “BiPella”, have the left ventricle supported by an Impella of varying capacities, such as 2.5/4/5.5 L/min, and the right ventricle supported by an Impella RP or with a Tandem Heart [3,4,5,6,7,8]. If the lungs need to be supported, an oxygenator can be added to any of the right-sided devices.

**Figure 2:**
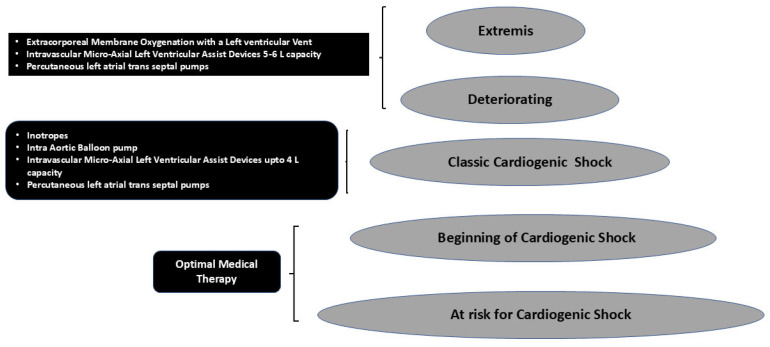
The utility of different treatment strategies used in increasing stages of severity of cardiogenic shock.

Figure 2 shows that as the stages of CS progress, more invasive therapies are needed to salvage the patient. Also when ECMO is used, invariably 30–70% of the patients have drastic increases in afterload, which would require unloading of the left ventricle for survival. However, unloading has its own costs in terms of monitoring for anticoagulation, vascular access sites and difficulty repositioning the patient. Hence, different combinations and permutations, as shown in Figure 1 and Figure 2, need to be used to tailor to each patient and each clinical situation to provide a personalized approach. The choice of mechanical unloading strategies/devices and their timing of insertion are usually dictated by individual treatment goals for an individual patient as well as local experience of the multidisciplinary team, which may be guided by scientific evidence. Many determinants that drive outcomes are largely based on clinical, hemodynamic and echocardiographic parameters, which may help with risk stratification. However, newer risk factor identification strategies are needed to improve delivery of personalized medicine.

### 3.2. Differences in Outcomes in Cardiogenic Shock Patients with HF

Patients with cardiogenic shock secondary to HF show differences in outcomes as compared to those with acute myocardial infarctions (AMI). CS patients with acute-on-chronic decompensated HF and those with acute coronary syndromes (ACS) appear to differ in their presentation and outcomes, suggesting the phenotypes in all these cases vary widely and need to be investigated further. Such differences in phenotypes of cardiogenic shock patients may in turn dictate different outcomes in patients presenting with acute-on-chronic HF and those with ACS despite similar hemodynamic and biomarker profiles. This observation suggests that risk factors and characteristics yet to be identified may play a major role in improving survival and outcomes.

Phenotypes of patients with cardiogenic shock due to end-stage HF show elevated filling pressures, reduced oxygen delivery, increased anaerobic metabolism and less metabolic acidosis [9].

Another observation in recent studies shows that higher numbers of ACS patients with CS receive temporary MCS, also suggestive of a distinct clinical phenotype. The mortality is higher in the ACS group with cardiogenic shock versus those with acute-on-chronic decompensated HF despite the higher utilization of temporary MCS (31% vs. 10%, respectively) [10]. The higher number of temporary MCS devices used was statistically significant (59% vs. 26%) among those with ACS and cardiogenic shock in the study by Jones et al. [10]. Existing risk factors may not account for the pathophysiological differences between the two groups. Such differences may suggest that the existing risk stratification is grossly inadequate to identify patients who actually need temporary MCS for optimal stabilization. Table 1 lists some of the recent studies on outcomes in patients with CS secondary to HF. A single-center study showed no difference in 1-year survival between CS secondary to AMI or HF. However, overall use of the surgical micro-axial pump Impella 5.0/5.5 decreased the use of vasoactive substances and increased end-organ perfusion [11]. Hong et al. showed that patients with CS secondary to HF had a better survival rate than patients with CS secondary to AMI [12]. In a recent study by Balder et al., a novel configuration of tMCS involving VA-ECMO and a micro-axial pump such as the Impella for LV venting was used, in which the 30-day mortality was 20%. About 30% of patients showed cardiac recovery, but this cohort was small, comprising of only 20 patients [13]. In a single-center observational study of 107 patients, the 4.5 year actuarial survival was 91% in bridge to transplant (n = 34), 79% in BT LVAD (n = 25) and 63% in the post cardiotomy group (n = 42) [14]. There still appears to be a wide variation in outcomes in the existing literature, showing that further investigations are needed to better define the different phenotypes.

### 3.3. Gender Differences in Survival and Outcomes

HF is a leading etiology for cardiogenic shock. Gender-specific differences in outcomes in this population are less well understood. Female patients in cardiogenic shock tend to have worse survival as compared to their male counterparts [3]. They also tend to receive fewer invasive cardiac interventions compared to males after adjusting for other confounding factors. Females in cardiogenic shock have higher rates of 30-day mortality as compared to males. Females have been shown to have a 10% higher mortality rate than males after adjusting for other factors. In another study, female patients with cardiogenic shock had a 38% 30-day survival compared to 50% for males. In-hospital mortality in females with acute coronary syndromes (ACS) as compared to females with HF (heart failure complicated by cardiogenic shock) had poor survival at discharge.

Female patients appear to be less likely to receive lifesaving treatments, like coronary angiography and revascularization with percutaneous coronary intervention (PCI), compared to men. Women with cardiogenic shock secondary to HF were also less likely to receive advanced surgical therapies, like orthotopic heart transplantation (OHT) or durable left ventricular assist device (LVAD) [15].

The reasons for these disparities are not fully elucidated and possibly include differences in clinical presentation, severity of shock and treatment strategies. Women in heart failure with cardiogenic shock appear to present with higher acuity than males and also appear to have higher rates of major bleeding and complications due to vascular access. Vascular access complications have been attributed to smaller blood vessels noted in females.

The impact of gender on outcomes for cardiogenic shock was primarily seen in the patients with heart failure in cardiogenic shock and not in patients in cardiogenic shock due to ACS. This observation was not uniformly noted in all studies reviewed. Females with heart failure appeared to be in severe cardiogenic shock, such as SCAI stages D and E, with worse survival at hospital discharge. They were also likely to receive less aggressive treatment [15].

Females experienced a higher incidence of vascular complications than males with a strong impact on their survival [15]. Females who survived cardiogenic shock had fewer vascular complications than those who did not survive cardiogenic shock. These findings suggest that the clinical trajectory of women in cardiogenic shock are different and there exists a need to identify new pathways to improve outcomes among women who experience cardiogenic shock secondary to decompensated heart failure [3]. Such a gender difference is not seen in all studies on women experiencing cardiogenic shock secondary to acute myocardial infarctions. Some studies report higher mortality in women in cardiogenic shock secondary to ischemic heart disease, while others have reported no significant gender differences in the women with cardiogenic shock secondary to acute myocardial infarctions [16,17,18,19,20,21,22,23]. Such differential observations may be due to study subject heterogeneity which dictates further investigations to differentiate the different phenotypes that may be influenced by different etiologies.

The difference in survival in women versus men presenting with cardiogenic shock are also due to an increased number of comorbidities, prolonged symptom duration from onset to first medical contact and delays in diagnosis and management. Additionally, women most often present with de novo CHF [24,25].

In an earlier analysis using Critical Care Cardiology Trials Network registry it was shown that overall patients with de novo heart failure had more severe shock presentation and worse in-hospital outcomes compared with those without heart failure, and women fair even worse than the males [24].

Female patients had more vascular complications, bleeding necessitating surgery and/or intervention and an increased incidence of limb ischemia as compared to males. Such observations have been noted in female patients undergoing coronary/structural interventions and percutaneous ventricular assist devices [26,27,28,29,30]. Increased vascular complications and acute limb ischemia occur in women due to smaller sized iliofemoral arteries than their male counterparts even when adjusted for BMI and the presence of peripheral arterial disease [31]. Female survivors of cardiogenic shock were those with fewer vascular complications [15]. Such gender-based differences noted after adjusting for confounding factors dictate the need for future studies to identify gender-specific risk factors. This is highlighted by the fact that hypertension, diabetes and history of coronary artery bypass grafting did not impact female patients in cardiogenic shock but were salient predictors of death in males, suggestive of different risk factors for mortality in males versus females. The use of pulmonary artery catheters improves survival in males, but the use of these catheters appears to be limited in females [15]. Future studies are needed to find gender-specific determinants of outcomes that appear to be different from what is known today.

Table 2 shows selected studies addressing gender-based differences in outcomes in female patients with CS secondary to HF. No differences in outcomes were noted in women in a study by Epps et al. No difference in in-hospital mortality in women with cardiogenic shock secondary to HF versus those secondary to AMI or as compared to their male counterparts [20] was noted. Fisher et al. showed in a meta-analysis that after adjusting for confounders, mortality for cardiogenic shock in females is 10% higher than their for their male counterparts [32]. The studies reviewed did not consistently show a gender-based difference suggestive of heterogeneity in the study population.

Some of the approaches to consider in improving outcomes in female cardiogenic shock patients would be to increase awareness of differences in response to standard treatments, develop temporary MCS devices suitable for the smaller vasculature in females, increase the number of females in clinical trials, develop registries exclusively for women in cardiogenic shock and develop robust risk prediction models with good discriminatory power using Artificial Intelligence (AI)-driven technologies.

### 3.4. Use of AI-Driven Technologies to Identify Risk Factors

Existing risk factors, such as renal failure, cardiac arrest, lactic acidosis and extent of requiring escalating doses of vasopressors, focus on mortality in patients who have developed CS. Hence, it is imperative to identify risk factors that predict CS early on in the pre-CS stage. Such early prediction of CS would alter the course of CS and possibly improve outcomes. The key to the early recognition of these early stages and initiating appropriate therapy requires efficient risk factor identification and robust risk prediction models.

In the current literature, the ORBI study prediction models seem to address CS secondary to AMI [33,34,35,36,37]. The importance of age, vital signs such as resting blood pressure, physical exam findings, biomarkers such as NT-proBNP, angiographic findings and revascularization success were used to predict the likelihood of CS secondary to AMI. Similar parameters without the revascularization/angiographic parameters have been used to predict CS secondary to HF. However, there seem to be differences in CS secondary to HF. Such differences tend to have implications in management and prognosis [38]. Another important factor to consider is that these risk factors do not accurately discriminate against the different CS phenotypes. The current risk factor research is largely driven by regression analysis. Regression analysis is useful in testing associations, but it is not very useful in prediction analysis and will not account for non-linear associations. Regression analyses may not be useful if the ratio of predictors to the number of effects is high [39].

Hence, it would be beneficial to use AI-driven technologies to investigate the different phenotypes. In machine learning models, the data are processed for the best fit in training, followed by validation and testing. Different AI-driven models have different pros and cons. Many methods need to be evaluated to arrive at the optimal models. One of the more promising models has been the XGBoost in predicting risk in CS. It has the ability to use scant data and allow parallel decision trees, avoids overfitting and is a model capable of handling non-linear inputs and outputs with a high precision. Others have used machine learning to phenotype CS secondary to AMI and HF [40,41,42,43]. Zweck et al. identified three different CS phenotypes using machine learning techniques [41]. Bai et al. used multiple methods, such as least absolute shrinkage and selection operator (LASSO), tree-based ensemble machine learning models, support vector regression (SVM) and (LightGBM) as well as XGB, to predict CS secondary to AMI [42]. Interestingly, linear models had a high predictive power (AUC = 0.92) [41]. The predictors included the time to first medical contact > 12 h for the development of CS.

Machine learning methods used to identify patients at higher risk for CS with acute decompensated heart failure included a study using EHR. The EHR data showing the high-risk group had a 10.2 times higher prevalence of developing CS in the following 24 h versus the low-risk group, but the positive predictive value was only 10% [42]. Hence, such models require further refinement and tailoring for individual patients and clinical scenarios.

Models should be developed from a generalized cohort so that it has substantial discriminatory power to evaluate a general hospitalized population at risk for CS development. Such an approach limits selection bias and allows generalizability. Models designed to continuously monitor electronic health records (EHR) data provide real-time data facilitating the mobilization of resources in a timely fashion. The model should have enough discriminatory capacity so that CS-secondary-to-HF patients can receive targeted disease-specific therapies.

Algorithms that recognize shock earlier and facilitate management may provide better outcomes. Such early predictive models could initiate CS-targeted therapies earlier. Practical applications of models should be tailored for every clinical situation and should trigger early alerts. When applied to individual patients, aggressive measures could be activated depending on the risk of developing CS and tailored to each specific patient and clinical situation. Efficient reliable models are instrumental in delivering personalized medicine with precision. Such models tend to have a meaningful impact on diagnosis and treatment. Finally, it is important that sensitivity and specificity of the models are balanced so that excessive false positives or negatives do not hamper the performance of the model.

### 3.5. AI-Driven Strategies to Improve Outcomes in Cardiogenic Shock Secondary to Heart Failure

**Figure 3:**
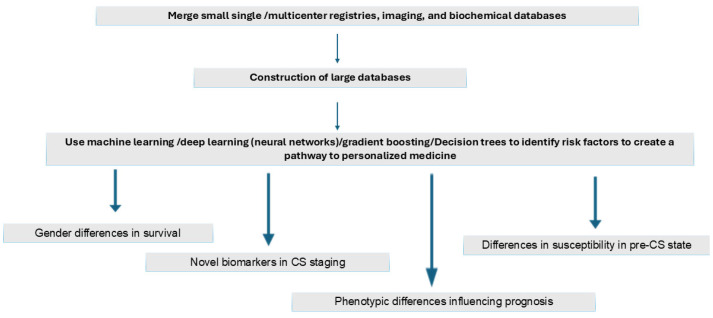
AI-driven strategies to improve outcomes in cardiogenic shock secondary to heart failure.

Figure 3 shows possible strategies to identify novel risk factors to identify CS at an early stage so that impending shock could be averted. The merging of single or multicenter databases containing images and laboratory data should help construct large databases on which many AI-driven/machine learning models such as neural networks and gradient boosting can work to identify novel risk factors to create effective risk prediction models and help pave the pathway to personalized medicine. Such models could possibly help discriminate the different CS phenotypes that have similar hemodynamic parameters. These approaches should help identify risk factors for gender differences in survival, arrive at a better staging system for pre-CS and identify phenotypic differences to help better prognosticate and finally define pathways that create differences in susceptibility in the pre-CS stage.

### 3.6. Use of Machine Learning in Improving the Management of CS Phenotypes

**Figure 4:**
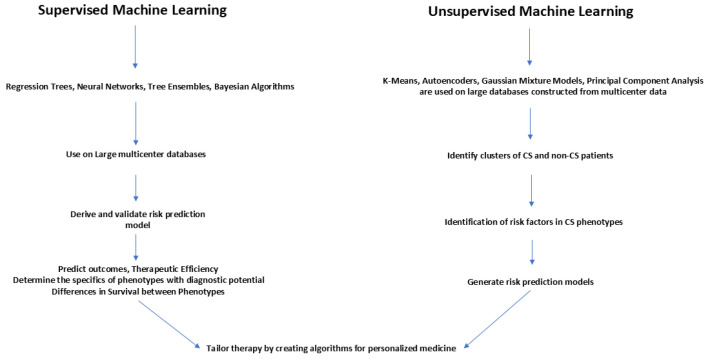
Use of machine learning in improving the management of CS phenotypes.

Figure 4 summarizes the use of machine learning in improving the management of CS phenotypes to generate AI-driven algorithms to identify CS phenotypes. Machine learning can be supervised or unsupervised. Supervised machine learning could be used on labeled data to train prediction models. Unsupervised machine learning uses unlabeled data to recognize patterns. Supervised learning is useful in classification and regression, and uses specific target variables for prediction. Unsupervised learning is used to detect hidden patterns in unlabeled data with no predefined outputs.

Large multicenter databases should be probed with supervised learning using regression trees, neural networks, tree ensembles and Bayesian algorithms to derive and validate risk prediction models to predict outcomes and the therapeutic efficiency of interventions and to determine the specifics of phenotypes for early diagnosis as well as for ascertaining survival differences between the phenotypes.

Unsupervised machine learning using k-means, autoencoders, Gaussian mixture models, and principal component analysis should be utilized to probe large databases constructed from multicenter data to identify clusters of CS and non-CS patients. These clusters can be further probed to identify sub-phenotypes with different risks. Risk factors thus identified can then be used to develop risk prediction models which can distinguish similar subtypes.

Therefore, by using supervised and unsupervised machine learning, large databases can be probed to derive and validate risk prediction models. By generating prediction models with high discriminatory power, subtle differences in sub-phenotypes can be identified so that tailored therapies can be made available for the delivery of personalized medicine [44,45].

In the existing literature, machine learning approaches were used in the International and Danish shock registries (Cardiogenic Shock Working Group Registry) (myocardial infarction) and acute-on-chronic heart failure registry and the Danish Retroshock MI Registry. This large cohort of 1959 patients consisted of both CS-due-to-myocardial-infarction and CS-due-to-heart-failure patients. K clustering was used in this study. Three clusters— “non-congested (I)”, “cardiorenal (II),” and “cardiometabolic (III)”—were identified. The initial identification of clusters was undertaken using the Cardiogenic Shock Working Group Registry) (myocardial infarction) and validated in the acute-on-chronic heart failure registry and the Danish Retroshock MI Registry. Interestingly, the cardiometabolic phenotypes or cluster had the highest rate of progression to stages D and E and also the highest in hospital mortality irrespective of the etiology [40].

### 3.7. Gaps in Knowledge

In the current clinical guidelines for tMCS, knowledge gaps exist in specifically tailoring timing and device selection based on individual patient acuity, characteristics and etiology of CS. Though guidelines exist, there is a need for more data-driven scientific evidence for recommendations, especially regarding the optimal time to initiate tMCS relative to other interventional therapies like revascularization. Additionally, the best device (whether IABP, Impella or ECMO) for the different etiologies underlying CS remains ambiguous. There is also a lack of specificity for devices for different etiologies. Optimal device selection strategies remain unclear in the existing literature. The data in the existing literature will need to be boosted with more robust clinical trials for the different etiologies driving CS. The existing classification of CS will need further refinement to guide device selection to tailor different CS etiologies. A multidisciplinary approach currently used is helpful but is very resource-intensive, making it challenging in some settings where adequately trained personnel and appropriate equipment may be lacking. There is a lack of definitive parameters for initiating the wean process from tMCS and the long-term care of these patients. The latest consensus guidelines based on expert consensus could be regarded as the first step in paving the way to more robust and definitive evidence-based guidelines [46].

## 4. Discussion

This review highlights the fact there is a clear lack of agreement in the literature regarding gender-based or etiology-based differences in outcomes for CS. In a small study from Spain using 164 patients with different etiologies, no differences in survival were noted with respect to gender differences. However, women constituted only 24% of the study population [22]. In another single-center study using CS patients with varied etiologies, no gender-based disparities in survival were noted. Interestingly, female patients who had percutaneous interventions showed a lower risk of 1-year mortality as compared to those who did not have interventions. It is unclear if this suggests interventions are used less as a mortality-reducing treatment in women [47].

It is also not clear if outcomes in CS are influenced by different etiologies. In CS due to acute myocardial infarctions, the culprit vessel revascularization is very important because this remains the basis of persistent hypotension and hypoperfusion. In CS secondary to HF, congestion and lack of perfusion and hypotension lead to a detrimental spiral. Hence, the takeaway from these observations is that tailoring of hemodynamic parameters using vasoactive medications and temporary MCS is essential to manage the different phenotypes, with different shock severity associated with each etiology [48].

The lack of randomized clinical trials and the fact that currently known risk factors do not differentiate the different existing phenotypes makes it difficult to achieve good outcomes or improve existing outcomes. Using AI-driven technologies to identify novel risk factors and applying a combination of machine learning and linear regression to develop new risk prediction models with robust discriminatory power would possibly open pathways to tailor treatment for different clinical scenarios that present with seemingly similar characteristics.

The most important goal for the future is to devise a methodology to understand and differentiate the different CS phenotypes and their trajectories. There is a need to include more women in clinical trials/studies and focus on the influence of gender and ethnic differences in outcomes. Better risk prediction models should be generated to help deliver efficient well-tailored treatment for different clinical scenarios, paving the way to the efficient delivery of personalized medicine.

## 5. Conclusions

Cardiogenic shock leading to circulatory collapse, hypoperfusion and end-organ dysfunction/damage carries a large burden of mortality, but management strategies are driven by expert consensus rather than adequately controlled clinical trials. This study highlights the differences in presentations and outcomes in cardiogenic shock depending on the etiology, such as acute myocardial infarction versus acute and chronic heart failure, gender-based differences in treatment strategies and outcomes. This may be achieved by identifying novel risk factors that differentiate the different phenotypes with similar hemodynamic and biomarker profiles and therefore help in early diagnosis. This study shows that there is a lack of consistency in the current literature on gender-based or ethnicity-based differences in outcomes in patients with CS and a lack of robust studies to define these knowledge gaps. There is a need to generate awareness of the dangers of CS among the female population and the necessity to make care accessible to female patients who appear to be presenting at later stages of CS, resulting in poorer outcomes. The study also points to an immediate need for more precise risk stratification and modeling to improve the efficiency of treatment delivery in a personalized fashion.

## Figures and Tables

**Table 1:** Selected studies on use of tMCS in CS secondary to HF.

Study	Study Type/Device Used	Number of Subjects N	End Point	Conclusions
George et al., 2023 [11]	Retrospective single-center using Impella 5.0/5.5	N = 90	1-year survival	No change in survival Improved end-organ perfusion Decreased need for vasoactive substances
Hong et al., 2024 [12]	Retrospective single-center using Impella 5.0/5.5	Total N = 137 AMI = 47 HF = 86 Post cardiotomy = 4	Survival to discharge	Survival to discharge was better in patients with CS secondary to HF
Balder et al., 2024 [13]	Retrospective study using ECPELLA	N = 20	30-day mortality	20% mortality at 30 days; 30% cardiac recovery
Mahesh et al., 2024 [14]	Impella 5.0/5.5 single-center observational study	N = 107	4.5 years	Actuarial survival was 91% in bridge to transplant (n = 34), 79% in BT LVAD (n = 25) and 63% in the post-cardiotomy group (n = 42)

**Table 2:** Selected studies on gender-based differences in CS outcomes.

Study	Study Type/Device Used	Number of Subjects N	End Point	Conclusions
Ton et al., 2023 [15]	Retrospective	5083	Survival at discharge	Women with CS secondary to HF had worse outcomes and more vascular complications than their male counterparts
Epps et al., 2023 [20]	Retrospective	520 (151 females and 369 males)	In-hospital mortality	No difference in women with cardiogenic shock secondary to HF versus those secondary to AMI as compared to their male counterparts
Fisher et al., 2024 [32]	Meta-analysis	656,754 females; 1,018,036 males	Combined in-hospital/30-day mortality	After adjusting for confounders, mortality for cardiogenic shock in females is 10% higher than for their male counterparts.

## Data Availability

No new data were created or analyzed in this study.

## Abbreviations

The following abbreviations are used in this manuscript:

CS	Cardiogenic shock
ACS	Acute coronary syndrome
tMCS	Temporary mechanical circulatory support
MCS	Mechanical circulatory support
